# Factors influencing symptom appraisal and help-seeking of older adults with possible cancer: a mixed-methods systematic review

**DOI:** 10.3399/BJGP.2021.0655

**Published:** 2022-08-23

**Authors:** Daniel Jones, Erica Di Martino, Stephen H Bradley, Blessing Essang, Scott Hemphill, Judy M Wright, Cristina Renzi, Claire Surr, Andrew Clegg, Richard Neal

**Affiliations:** Leeds Institute of Health Sciences, University of Leeds, Leeds.; Leeds Institute of Health Sciences, University of Leeds, Leeds.; Leeds Institute of Health Sciences, University of Leeds, Leeds.; Leeds Institute of Health Sciences, University of Leeds, Leeds.; Leeds Institute of Health Sciences, University of Leeds, Leeds.; Leeds Institute of Health Sciences, University of Leeds, Leeds.; University College London, London.; Leeds Beckett University, Leeds.; Academic Unit for Ageing & Stroke Research, University of Leeds, Leeds.; School of Medicine, University of Leeds, Leeds.

**Keywords:** early detection of cancer, frail elderly, primary health care, systematic review

## Abstract

**Background:**

The cancer burden falls predominantly on older (≥65 years) adults. Prompt presentation to primary care with cancer symptoms could result in earlier diagnosis. However, patient symptom appraisal and help-seeking decisions involving cancer symptoms are complex and may be further complicated in older adults.

**Aim:**

To explore the effect of older age on patients’ appraisal of possible cancer symptoms and their decision to seek help for these symptoms.

**Design and setting:**

Mixed-methods systematic review.

**Method:**

MEDLINE, EMBASE, CINAHL, PsycINFO, Cochrane Library, Web of Science Core Collection, ASSIA, the ISRCTN registry, and the National Institute for Health and Care Excellence were searched for studies on symptom appraisal and help-seeking decisions for cancer symptoms by adults aged ≥65 years. Studies were analysed using thematic synthesis and according to the Synthesis Without Meta-Analysis guidelines.

**Results:**

Eighty studies were included with a total of 32 995 participants. Studies suggested a possible association between increasing age and prolonged symptom appraisal interval. Reduced knowledge of cancer symptoms and differences in symptom interpretation may contribute to this prolonged interval. In contrast, in the current study a possible association was found between increasing age and prompt help-seeking. Themes affecting help-seeking in older adults included the influence of family and carers, competing priorities, fear, embarrassment, fatalism, comorbidities, a desire to avoid doctors, a perceived need to not waste doctors’ time, and patient self-management of symptoms.

**Conclusion:**

This review suggests that increasing age is associated with delayed cancer symptom appraisal. When symptoms are recognised as potentially serious, increasing age was associated with prompt help-seeking although other factors could prolong this. Policymakers, charities, and GPs should aim to ensure older adults are able to recognise potential symptoms of cancer and seek help promptly.

## INTRODUCTION

Worldwide, the population of adults aged >65 years is growing faster than any other age group.^1^ The burden of cancer falls predominantly on older patients with half of all new diagnoses occurring in people aged >70 years and incidence rates for all cancers increasing most rapidly in the >75 years age group.^1^^,^^2^

Shorter times to diagnosis result in more favourable cancer outcomes and patient experiences.^3^^,^^4^ As a result, cancer strategies prioritise prompt presentation by patients with symptoms that could be caused by cancer. However, previous qualitative research has highlighted the complexity of patient appraisal and help-seeking decisions involving cancer ‘alarm symptoms’. This complexity includes a lack of awareness of cancer symptoms as well as concerns about wasting doctors’ time and fears around facing a diagnosis of cancer, all of which may delay presentation to primary care.^5^^,^^6^ In older adults, the recognition of cancer symptoms could be further complicated by the presence of chronic diseases and cognitive impairment that may affect the ability to recognise new symptoms.^7^^,^^8^ In addition to this, symptoms may be dismissed as part of the ageing process and social isolation may result in the absence of a person to prompt presentation.

The aim of this review is to explore the effect of older age on the appraisal of symptoms that could indicate cancer in older adults and the decision to seek help in primary care for these symptoms.

## METHOD

### Protocol

Before commencing this review, a study protocol was registered with PROSPERO (reference number: CRD42020180656). The review has been conducted and reported according to the Cochrane Handbook for Systematic Reviews and the Preferred Reporting Items for Systematic Reviews and Meta-Analyses (PRISMA) statement.^9^

### Definition of older adults

There is no universally accepted age threshold for defining old age. The World Health Organization’s definition of ‘older people’ as those aged ≥65 years was adopted.^10^

### Theoretical model

The model of pathways to treatment was used to provide a theoretical underpinning for the analysis of patient appraisal and help-seeking.^11^ The model is recommended for mapping and examining pathways to cancer diagnosis. The model defines the appraisal interval as the time between detecting a bodily change and perceiving a reason to discuss this bodily change with a healthcare professional (HCP). The help-seeking interval is defined as the time between perceiving a need to seek medical help and arranging and attending an appointment with an HCP. The patient interval encompasses both the appraisal and help-seeking interval, and is defined as the time from first noticing a symptom to presenting to an HCP.

**Table:** How this fits in

The burden of cancer falls predominantly on older (≥65 years) adults, and prompt presentation to primary care with cancer symptoms results in better patient outcomes. The current review, which included 80 studies, found that older adults with cancer symptoms may have prolonged symptom appraisal and shorter help-seeking intervals prior to presenting to general practice. Factors such as knowledge of cancer symptoms, the influence of family and carers, fear, embarrassment, comorbidities, and patient self-management all affected the appraisal or help-seeking interval. Clinicians should be aware of patient difficulty in distinguishing potentially worrying cancer symptoms from symptoms of ageing as a result of frailty or comorbidities.

### Eligibility criteria

Studies of patients aged ≥65 years, or with a subgroup of patients aged ≥65 years, with symptoms and signs that warrant investigation and referral for suspected cancer^12^ presenting to primary care before diagnosis were included in the current study. Editorials, case studies, reviews, expert opinion papers, and studies that were published as abstracts only were excluded from the review. Systematic reviews, published theses, and dissertations were not included but when these were identified reference lists were screened for relevant studies.

### Search strategy

Ovid MEDLINE(R) ALL 1946 to 28 July 2021, EMBASE (Ovid) 1947 to 28 July 2021, CINAHL (EBSCOhost), APA PsycINFO (Ovid) 1806 to week 3 of July 2021, Cochrane Central Register of Controlled Trials (Wiley) Issue 7 of 12, July 2021, Cochrane Database of Systematic Reviews (Wiley) Issue 7 of 12, July 2021, Web of Science Core Collection (individual databases are listed in Supplementary Information S1), Applied Social Sciences Index and Abstracts (ProQuest), ISRCTN registry, ClinicalTrials. gov, and the National Institute for Health and Care Excellence Evidence Search for published and unpublished studies of cancer-related shared decision making for older adults in primary care were searched. The search was originally conducted in April 2020 and updated on 29 July 2021 by re-running all searches without date limits and removing duplicates from the original searches using EndNote.

Subject headings and free-text words were identified for use in the search concepts by two authors and was based on a search strategy published in a similar review.^13^ No language, data, or study design limits were used. Full search strategies are available in Supplementary Information S1.

Further relevant studies were sought by reviewing reference lists of the included studies, and manually searching conference abstracts using the conference handbooks from events organised by the Cancer and Primary Care Research International Network,^14^ the National Cancer Research Institute,^15^ Macmillan Cancer Support,^16^ and Cancer Research UK.^17^

Search results were managed in an EndNote library where duplicates were removed automatically and manually using University of Leeds Academic Unit of Health Economics guidance.

### Data collection

All titles and abstracts were independently reviewed by two researchers using the review software Rayyan. Any disagreements were resolved through discussion or through adjudication by a third reviewer if required. All reasons for exclusion were recorded. Data extraction was undertaken using a data extraction template.

### Risk of bias of included studies

The mixed-methods appraisal tool (MMAT) was used to appraise the methodological quality of the included studies.^18^ The tool allows the appraisal of randomised, non-randomised, quantitative descriptive, qualitative, and mixed-methods studies. The tool provides five methodological quality criteria that vary for each type of study.^19^ The reviewers’ reasons for ratings, including strengths and weaknesses of studies, were recorded independently by two reviewers. Any disagreements were resolved through discussion.

### Synthesis of results

Qualitative studies were analysed using thematic synthesis described by Thomas and Harden.^20^ Thematic synthesis includes line by line coding of all results, the organisation of the codes into themes, and finally further interpretation to develop analytical themes that aim to offer a new interpretation of the study findings. This was undertaken independently by two reviewers. The ENTREQ guidelines for reporting the synthesis of qualitative research were followed.^21^

Quantitative studies did not provide data suitable for meta-analysis because of heterogeneous effect measures and clinical and methodological diversity. As a result, the analysis reporting was undertaken using the SWiM (Synthesis Without Meta-Analysis) reporting guidelines and checklist.^22^

Following the separate analysis of qualitative and quantitative data, the findings were combined by considering the barriers and facilitators to appraisal and help-seeking. This method was based on previous published guidance on integrating qualitative research in systematic reviews.^23^

## RESULTS

**Figure 1.**
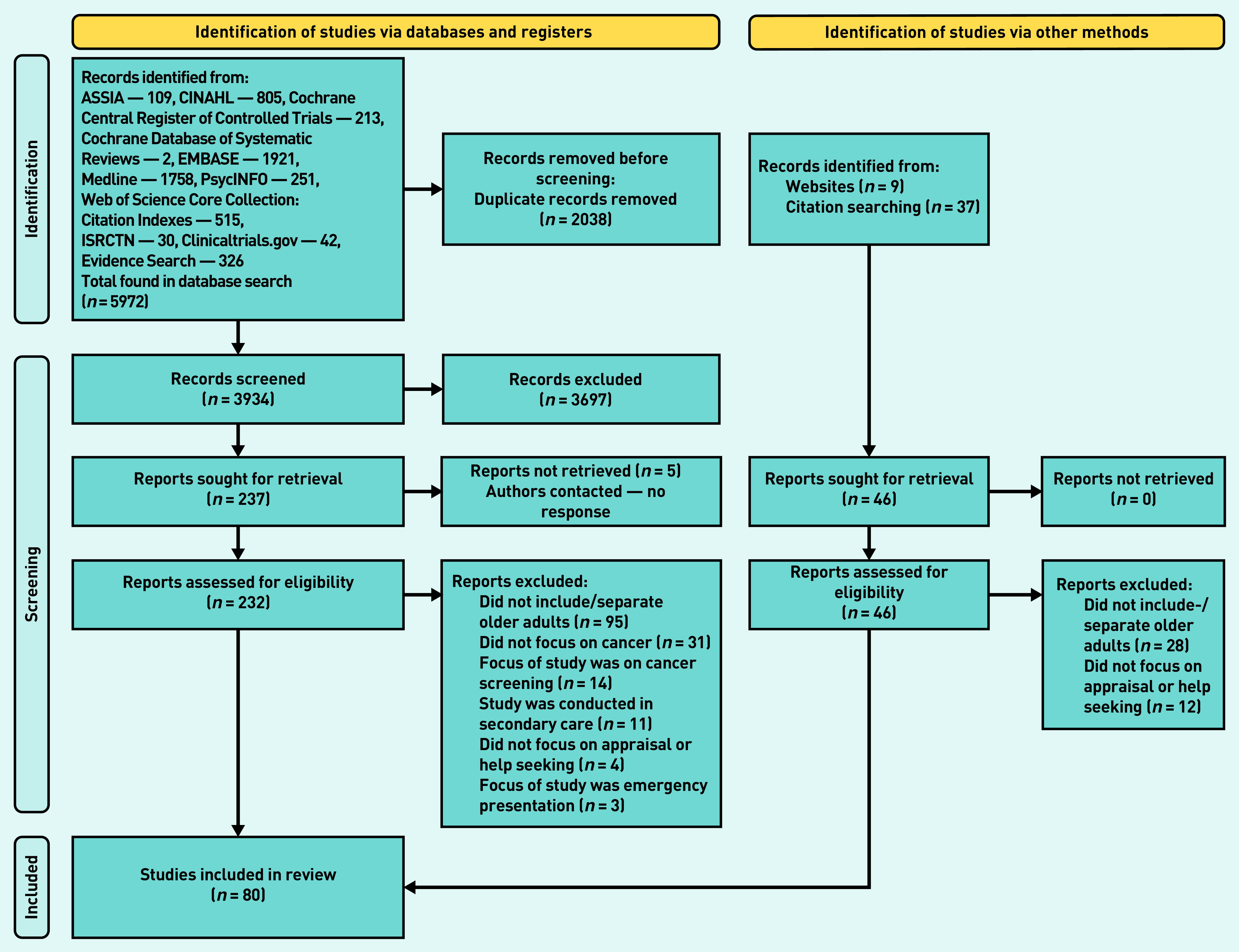
*PRISMA flow diagram.*

The database searches identified 5972 studies that reduced to 3934 when duplicates were removed. After title and abstract screening and full-text review, 80 papers were included in this review including 324 995 participants (Figure 1).

Studies ranged in size from 10 to 65 192 participants. Forty-six papers included quantitative data^24^^–^69 and 31 provided qualitative data,^70^^–^100 three were mixed-methods studies and provided both qualitative and quantitative data.^101^^–^103 Study settings were UK, Australia, Denmark, Estonia, France, Germany, Holland, Malaysia, South Africa, Spain, Sweden, Trinidad and Tobago, and the US.

**Figure 2.**
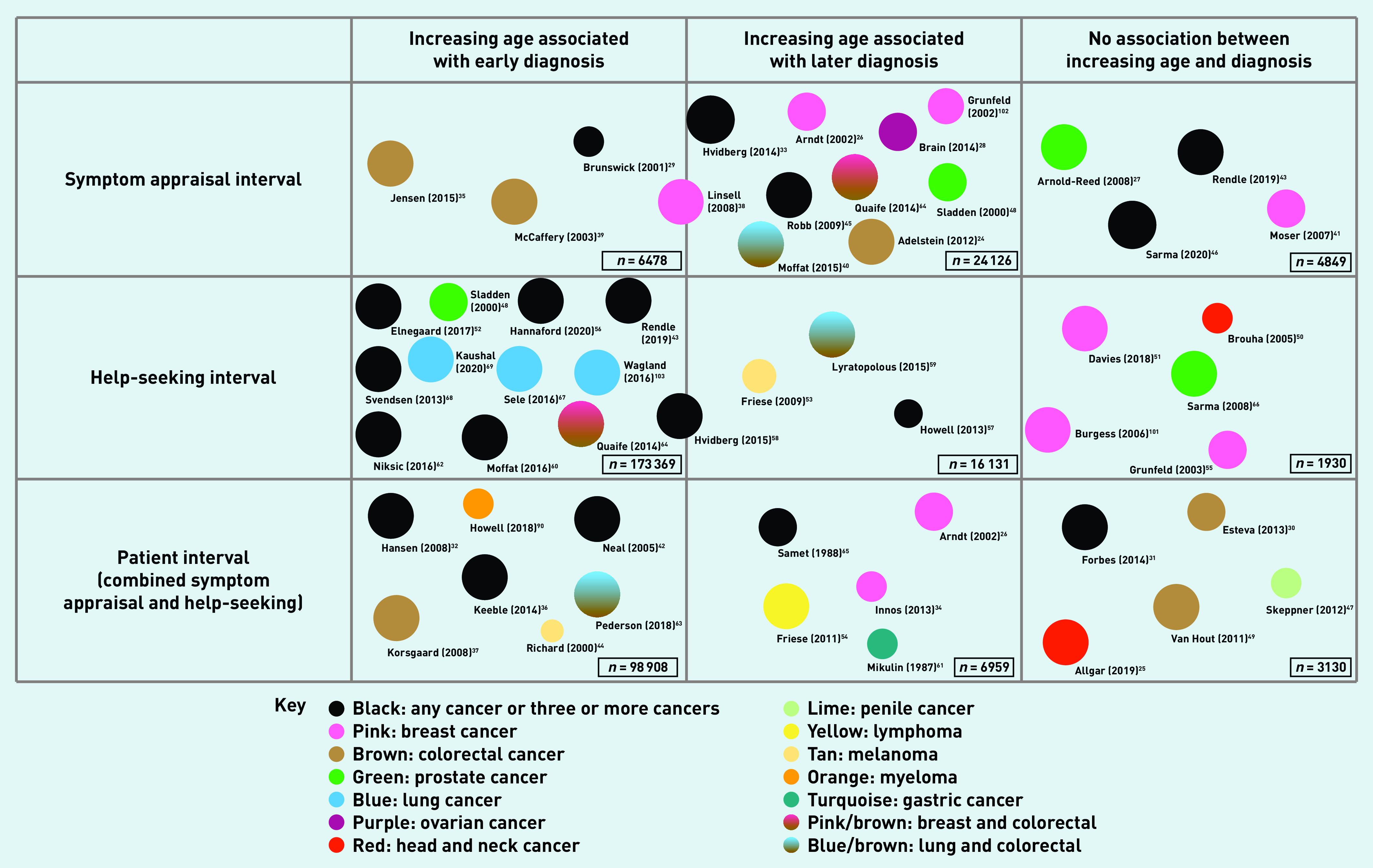
*Diagram to show the number of quantitative studies, the association with appraisal and help-seeking, the cancer investigated, and the quality assessment. Size of circle corresponds to the quality rating judged using MMAT. MMAT = mixed-methods appraisal tool.*

There were no clear differences in the appraisal or help-seeking of older adults by country and, although most of the included countries have a GP gatekeeper healthcare system, no system factors were highlighted in the studies. A variety of cancers were studied including ‘any cancer’, colorectal, brain, breast, lung, prostate, lymphoma, ovarian, upper gastrointestinal, bladder, cervical, leukaemia, gastric, myeloma, head and neck, melanoma, pancreatic, and penile. Overall, the quality of studies was judged to be high with an average MMAT across the 80 included studies of 4.6 out of a maximum possible score of 5.0. See Figure 2 for a summary of the results and Supplementary Tables S1–3 for details on the included studies.

### Appraisal interval

This review included 50 studies that considered the association between age and help-seeking for cancer symptoms. Twenty studies provided quantitative data. The majority of these studies suggest an association between increasing age and a shorter help-seeking interval. Ten studies found that increasing age was associated with shorter help-seeking interval,^43^^,^^48^^,^^52^^,^^56^^,^^60^^,^^62^^,^^67^^–^69^,^^103^ five studies found no association between age and help-seeking,^50^^,^^51^^,^^55^^,^^66^^,^^101^ and three studies found that increasing age was associated with a prolonged help-seeking interval.^53^^,^^57^^,^^59^ Two studies had mixed findings, highlighting factors that both shortened and prolonged the appraisal interval.^38^^,^^39^ These data are summarised in Supplementary Table S1.

Analysis of the 28 qualitative studies^70^^–^94^,^^99^^,^^101^^,^^102^ highlighted three themes affecting patients’ appraisal of possible cancer symptoms:

- symptom awareness;
- symptoms interpreted as old age; and
- symptoms interpreted as being caused by comorbidities.

The studies suggested that older adults were less aware of potential cancer symptoms resulting in patients not perceiving a need to present to an HCP. When a bodily change was detected, there were examples of patients explaining symptoms as part of the ageing process and as such did not perceive the bodily change as a reason to visit an HCP. Similarly, older adults with existing comorbidities tended to explain new bodily changes as being part of their existing medical problems, or as side effects of medication. This interpretation or normalisation of cancer symptoms resulted in patients not perceiving a need to consult with an HCP, and delaying diagnosis. Box 1 shows the themes from the qualitative analysis and illustrative quotes supporting these findings.

**Box 1.** Themes and illustrative quotes demonstrating the impact of age on the appraisal of symptoms,highlighting symptom awareness and interpretation

**Theme**	**Sub-theme**	**Illustrative quotes**
**Symptom awareness**	Studies suggested older adults are less aware of potential cancer symptoms	A man in the 65-to 69-years age group with a delayed diagnosis of colorectal cancer stated: *‘I wasn’t thinking of cancer, I’d never associated that sort of symptom with cancer.’* 79 A 75-year-old man with prostate cancer who delayed help-seeking for 6 months stated: *‘I would never have thought there’s something wrong with the prostate, I didn’t even know what the prostate was.’* 82 A 68-year-old man with persistent chest pain stated: *‘I’d never heard anything about checking for lung cancer or that type of thing.’* 83
**Symptoms interpreted**	Older adults tended to explain new symptoms as part of the ageing process	On discussing worsening breathlessness a patient stated: *‘I was not able to walk as fast as I used to be able to, but I didn’t think much about it. I am 72 I am going to begin to slow down.’* 77 Another patient noticed a breast change and stated: *‘I thought it was my age, sagging, shrinkage, you know, everything going south, as it were.’* 101 A patient eventually diagnosed with myeloma attributed multiple symptoms to the ageing process: *‘It was a gradual process which I put down to old age, I had a bad back, but I was willing to accept that my back hurt a bit.’* 90
**Symptoms interpreted as being caused by comorbidities**	Older adults tended to explain new symptoms as being a result of an existing comorbidity	A 69-year-old patient diagnosed with lung cancer states: *‘… yeah, well I’m asthmatic, you have a tendency to cough more.’* 76 A 73-year-old woman with colorectal cancer blamed her change in bowel habit on new cholesterol medication: *‘… she* [GP] *changed me to simvastatin and it was a while after I got these sensations, I thought is it because I am taking these statins?’* 76 As comorbidities and, as a result, medications increase with increasing age, this is more likely to affect older adults. A qualitative study of GP case reports of patients with lung cancer found a number of examples of comorbidities delaying diagnosis in older adults, including persistent coughing thought to be because of blood pressure medication and increased breathlessness thought to be because of heart failure.^96^

### Help-seeking interval

This review included 48 studies that considered the association between age and help-seeking for cancer symptoms. Eighteen studies provided quantitative data. The majority of these studies suggest an association between increasing age and a shorter help-seeking interval. Ten studies found that increasing age was associated with shorter help-seeking interval,^43^^,^^48^^,^^52^^,^^56^^,^^60^^,^^62^^,^^67^^–^69^,^^103^ five studies found no association between age and help-seeking,^50^^,^^51^^,^^55^^,^^66^^,^^101^ and only one found that increasing age was associated with a prolonged help-seeking interval.^59^ Two studies had mixed findings highlighting multiple factors that could both shorten and prolong the help-seeking interval. For example, one study found older adults were statistically less likely to want to know they had cancer (which may delay help-seeking), but were also less likely to be ‘put off’ by perceived barriers to help-seeking (which may shorten help-seeking).^58^^,^^64^ These data are summarised in Supplementary Table S2.

Four quantitative studies^59^^,^^60^^,^^62^^,^^64^ and 30 qualitative studies^70^^–^91^,^^95^^–^101^,^^103^ considered barriers to help-seeking for cancer symptoms among older adults. The studies highlighted and investigated several barriers to help-seeking that are listed below:

- the influence of family, carers, and friends (for example, older adults being encouraged to seek help by family or carers);
- competing priorities (for example, caring for unwell or frail spouses);
- fear (for example, fear of a cancer diagnosis or of investigations);
- the perceived role of the doctor in help-seeking (older adults perceiving ‘the doctor’ in a way that delayed help-seeking);
- comorbidities (conditions or diseases that tend to increase with advancing age); and
- self-management of symptoms (for example, ‘watchful waiting’ or trying ‘over-the-counter’ or alternative treatments before visiting the GP).

These themes and supporting illustrative quotes are presented in Box 2.

**Box 2.** Themes and illustrative quotes demonstrating the impact of age on patient help-seeking

**Theme**	**Sub-theme**	**Illustrative quotes**
**The influence of family and carers**	Family and carers encouraged older patients to seek help	A patient who was an ex-smoker in the 75-to 79-years age group and who had a persistent cough stated: *‘My wife persuaded me to go to the doctor about it. I wasn’t too worried about it.’* 77 A patient in the 65-to 69-years age group with symptoms suggested of colorectal cancer reported that her daughter was *‘on the bossy side’* and had said to her *‘don’t leave it mam, go.’*^79^
**Competing priorities**	Older adults reported delaying help-seeking as a result of competing priorities such as caregiving roles, or other life events	An older adult reported finding a breast lump but delayed seeking help because of her husband. She stated: *‘… my husband went into hospital for a hip operation. I thought I’dwait until he came home.’* 101 A 69-year-old male with symptoms of lung cancer was due to go on holiday and as a result did not report his symptoms so as to not *‘spoil the vacation’*.^75^
**Fear**	Older adults reported that fear and embarrassment resulted in a delay in help-seeking for cancer symptoms	A 71-year-old patient described deferring help-seeking because of ‘fear’: *‘I suppose deep down I didn’t want to know anything else, you know, we’re back to this thing that you know I think we are frightened, we do get frightened occasionally that … there is something more serious and so if you can sort of, pretend that it’s just a bad back, you’re quite happy to just accept that.’* 90 A patient in the 65-to 69-year age group reported the embarrassment of an intimate examination: *‘it was just the embarrassment of knowing that I might have to have somebody’s finger pushed up your bum for an examination … that was probably the thing that put me off going more than anything in the first instance was the embarrassment of that sort of thing.’* 79
**The perceived role of the doctor in help-seeking**	Older adults perceived ‘the doctor’ in a way that delayed help-seeking	An older patient reported: *‘I’m not someone who goes to the doctor. I never used to trouble. If I had a cold I’d see to it myself. I didn’t like the idea of going to be honest and I didn’t want to go. When I did go she said “Oh you’ve not been here for so many years”!’* 8 An older adult reported being embarrassed by seeing younger doctors: *‘I was beginning to come to my senses and thought I’d better go the doctors, but I’m an embarrassed person, you know, showing yourself like that … he’s a young, good-looking doctor.’* 101
**Comorbidities**	As a result of comorbidities, patients expect new symptoms and do not seek help	A 67-year-old woman with unexplained weight loss also had comorbidities that resulted in frequent ‘aches and pains’. As a result she reported only attending the doctor with serious symptoms: *‘I have the arthritis, and I have so many aches here, there, and everywhere. And the GP, there’s nothing he can do. I am already taking medications for that through the hospital, you know. So I don’t bother the GP with all my symptoms every time, just what I think is pertinent.’* 100
**Self-management**	Older adults described a preference for ‘watchful waiting’ or self-management that delayed help-seeking	A man in the 75-to 79-years age group described trying medication they could buy before seeking help: *‘You do the usual, you take your Lemsip’s and your Beechams Powders and when it doesn’t clear up after a week you think well you need some antibiotics or something slightly stronger. So that was when I went to the doctors.’* 77 A 71-year-old man with cancer symptoms stated: *‘I believed that this sickness was a sign for me to get right with God. I put off my baptism for a long time now and I realized that was what I needed to do. So I went and get baptised.’* 93

### The patient interval

A further 16 studies considered the association between increasing age and both appraisal and help-seeking intervals combined; this is known as the patient interval. These studies do not suggest an association between increasing age and the patient interval. Seven studies found that increasing age was associated with a shorter patient interval,^32^,^36^,^37^,^42^,^44^,^63^,^90^ five found increasing age had no effect on the patient interval,^25^^,^^30^^,^^31^^,^^47^^,^^49^ and four studies suggested patient age was associated with a longer patient interval.^26^^,^^34^^,^^54^^,^^61^ These data are summarised in Supplementary Table S3 while details of studies providing qualitative data are summarised in Supplementary Table S4.

## DISCUSSION

### Summary

Cancer is a disease of older adults with over half of new cancer diagnoses occurring in those >70 years of age. Decisions on the recognition and referral of cancer in older adults are difficult, with factors such as frailty and comorbidities meaning that management options are often limited. However, delayed presentation to primary care is likely to reduce the chance of curative treatment still further. If cancer symptoms are not recognised and help not sought, a large burden of potentially curable disease will be missed. As a result, this review has considered the effect of age on the presentation to primary care and the factors that affect this.

The findings of the review suggest an association between increasing age and a prolonged appraisal interval. As a result, the time from first noticing a bodily change to perceiving a need to seek help may be longer in older adults. In contrast, the review suggests an association between increasing age and a shortened help-seeking interval, with most studies suggesting older adults were more likely to seek help promptly when they detected symptoms that they perceived could be cancer. Despite this, the evidence highlighted several factors specific to older adults that could potentially delay help-seeking. Overall increasing age showed no association with the length of the patient interval.

### Strengths and limitations

To the authors’ knowledge, this is the first systematic review to investigate the association between increasing age and appraisal and help-seeking for cancer symptoms. This review includes 80 studies of good methodological quality and has been conducted in accordance with best-practice guidelines. Studies were included from a variety of countries and investigated a wide range of cancer types using both qualitative and quantitative methods.

This study, however, has some limitations. First, the heterogeneity of included studies precluded meta-analysis of quantitative data. Second, because of the observational nature of some of the included studies, it is unclear whether there is a causative link between older age and the factors highlighted. Finally, although all the data analysed was from those aged ≥65 years, there was little comparison between younger and older adults. As a result, some of the factors identified may not be specific to older adults and may be equally relevant to a younger patient group. All patients may, for example, have competing priorities and may experience emotions such as fear and embarrassment. It is possible that there are differences in the competing priorities and emotions of older adults such as perhaps being more likely to have dependent partners. These differences would not be possible to identify without further work to compare findings in older and younger adults.

Although many of the included studies were clearly investigating appraisal or help-seeking intervals, there were studies in which it was more difficult to disentangle the two intervals. This is challenging and a recognised methodological issue when exploring the pathway to cancer diagnosis.

### Comparison with existing literature

This review suggests an association between increasing age and prolonged symptom appraisal. The qualitative studies in this review suggested that a lack of cancer knowledge and normalising symptoms may contribute to this. Earlier studies support this finding, suggesting that older adults have lower cancer awareness measure scores^104^ and lower health literacy.^105^

Factors affecting help-seeking in this review were also highlighted in other studies. In one review older adults cited family and carer difficulties^106^ as a barrier to seeking help for symptoms of dementia. A review on the impact of comorbidities on cancer diagnosis found that comorbidities could be associated with both prolonged and shortened time to diagnosis,^107^ supporting the finding of this review. Fear was a prominent theme in a review considering help-seeking for cancer symptoms in all age groups.^108^ A systematic review considering the risk factors for delayed presentation of cancers found evidence of delay in older patients with breast cancer and reported that fear of cancer contributed to this delayed presentation.^5^

Studies have considered factors affecting appraisal and help-seeking in the general population. One study found that symptom knowledge and correct symptom interpretation improved the appraisal interval in the general population, supporting the findings of this study.^109^ Whitaker *et al* reported similar factors to this review, in their study on adults aged ≥50 years but also report others such as lack of confidence in the healthcare system and the importance of ‘instinct’ or gut feeling that were not found in this current review.^6^

### Implications for research and practice

This review suggests that increasing age is associated with prolonged appraisal of cancer symptoms and a shortened time to seek help. Symptom appraisal in older adults is complex, with multiple issues such as a possible lack of awareness of cancer symptoms, the attribution of symptoms to ageing, and the presence of other comorbidities. Further research needs to investigate the best approaches to improve symptom appraisal in older adults. It is possible that cancer awareness campaigns, such as the UK’s ‘Be Clear on Cancer’^110^ and the US Centers for Disease Control’s ‘Inside Knowledge’,^111^ may improve awareness; however, subsequent cancer awareness campaigns should be designed to ensure they reach an older population through more targeted advertising or through other means such as cancer champions in day centres for older people or care homes.

The challenge of distinguishing symptoms that may be because of cancer from normal signs of ageing and symptoms of comorbidities is complex and difficult. In primary care, older adults are more likely to have contact with primary care because of the higher burden of comorbidities in this population. It is possible that annual reviews of other chronic conditions may allow an opportunity for HCPs and patients to recognise and discuss cancer symptoms. Posters or literature in waiting rooms could also support older adults to recognise cancer symptoms. When patients are diagnosed with chronic conditions there is an opportunity to discuss and advise patients about expected symptoms and their duration, and symptoms that should prompt concern.

Although increasing age was possibly associated with a shortened help-seeking interval, this review highlighted potential barriers to help-seeking in older adults. Future research could be targeted at interventions to reduce the impact of these barriers. Healthcare practitioners and policymakers may be able to implement changes to encourage patients with cancer symptoms to present promptly. For example, this current review found that a dislike of visiting GPs and a fear of wasting doctors’ time may delay help-seeking. Local or national campaigns could seek to legitimise help-seeking in older adults. This may include public health information that symptoms should not be normalised or attributed to age, on the expected time course or length of symptoms, and the need to return if symptoms persist. In light of the COVID-19 pandemic and the changes this has made to general practice, public information targeted at older adults on the safety of attending general practice, the availability of GPs, and how best to access care may be helpful.

The management of fatalistic attitudes is difficult, but incorporating information about the curability of early-stage cancers in public education campaigns may improve help-seeking.^59^ Although self-management is encouraged by policymakers, the current review suggests an unintended consequence of this self-management may be delayed presentation with symptoms. Pharmacists and dispensaries could be well placed to recognise potentially harmful self-management of certain red-flag symptoms (such as the use of antacid medication for dyspepsia) and are encouraged to promote help-seeking; however, there are no formal referral pathways for community pharmacists to refer patients to primary care or any mechanism for them to ensure help-seeking has taken place.^112^ Further research is needed and future public health self-management messages may need to take this into account, especially in older adults with potential red-flag symptoms. Finally, the role of family and carers was highlighted in this current review;= increasing social isolation in older people is well documented but the effect of this on cancer diagnosis is unknown and is a key area of future research.

With increasing age comes an increasing risk of other factors such as frailty syndromes, comorbidities, and cognitive impairment that may confound the results of this review. It is possible that these factors may play a significant role in the appraisal and help-seeking related to cancer symptoms and are as or more important than age alone. Future research should try to disentangle this, and explore the effect that comorbidities, frailty, and cognitive impairment have on cancer diagnosis.

## Data

The full search strategy and all data are available in the supplementary material.
